# Mir-190b negatively contributes to the *Trypanosoma cruzi-* infected cell survival by repressing PTEN protein expression

**DOI:** 10.1590/0074-02760150184

**Published:** 2015-12

**Authors:** Cíntia Júnia Monteiro, Suianne Letícia Antunes Mota, Lívia de Figueiredo Diniz, Maria Terezinha Bahia, Karen CM Moraes

**Affiliations:** 1 Universidade Federal de Ouro Preto, Instituto de Ciências Exatas e Biológicas, Núcleo de Pesquisa em Ciências Biológicas, Departamento de Ciências Biológicas, Laboratório de Doença de Chagas, Ouro Preto, MG, Brasil; 2 Universidade Estadual Paulista Júlio de Mesquita Filho, Instituto de Biociências, Departamento de Biologia, Laboratório de Biologia Molecular, Rio Claro, SP, Brasil

**Keywords:** cardiac cellular model, microRNA, PTEN, Trypanosoma cruzi

## Abstract

Chagas disease, which is caused by the intracellular protozoan*Trypanosoma cruzi*, is a serious health problem in Latin America. The heart is one of the major organs affected by this parasitic infection. The pathogenesis of tissue remodelling, particularly regarding cardiomyocyte behaviour after parasite infection, and the molecular mechanisms that occur immediately following parasite entry into host cells are not yet completely understood. Previous studies have reported that the establishment of parasitism is connected to the activation of the phosphatidylinositol-3 kinase (PI3K), which controls important steps in cellular metabolism by regulating the production of the second messenger phosphatidylinositol-3,4,5-trisphosphate. Particularly, the tumour suppressor PTEN is a negative regulator of PI3K signalling. However, mechanistic details of the modulatory activity of PTEN on Chagas disease have not been elucidated. To address this question, H9c2 cells were infected with *T. cruzi* Berenice 62 strain and the expression of a specific set of microRNAs (miRNAs) were investigated. Our cellular model demonstrated that miRNA-190b is correlated to the decrease of cellular viability rates by negatively modulating PTEN protein expression in *T. cruzi*-infected cells.

Chagas disease, which is caused by the intracellular protozoan pathogen*Trypanosoma cruzi*, is a leading cause of cardiomyopathy and heart failure in Latin America. More than eight million people are estimated to be affected by this parasite and thousands of others are at risk of infection (WHO 2013). *T. cruzi* induces multiple responses in the heart, which is a critical organ that is involved in infection and host pathology (Zhang & Tarleton 1999). The initial cardiomyocyte response to *T. cruzi* is fundamental to establish the heart infection and is dependent on the activation of host cell signalling pathways that involve the immediate activation of kinases and phosphatases as well as changes in gene expression patterns (Burleigh & Woolsey 2002,Burleigh & Soldati 2008). The complexity of *T. cruzi* cellular invasion and survival has been considered a challenge. The understanding of such molecular mechanisms could contribute to the development of new therapies, because curing this disease is possible if treatment is initiated soon after infection.

Over the last decade, research to clarify cardiomyocyte behaviour after parasitic infection has been conducted (Petersen & Burleigh 2003, Calvet et al. 2012, Corral et al. 2013). Some studies reported the activation of the phosphatidylinositol-3 kinase (PI3K)/Akt pathway in *T. cruzi* infected cells (Chuenkova et al. 2001, Aoki et al. 2004) and this activation contributes to host cell survival or death. PI3K controls important steps in cellular metabolism by regulating the production of the second messenger phosphatidylinositol-3,4,5-trisphosphate (PIP3), which is able to activate AKT and downstream signalling events such as heart hypertrophy, cellular proliferation, and even apoptosis (Maehama & Dixon 1998). In particular, the tumour suppressor PTEN is a negative regulator of PI3K signalling, because it hydrolyses PIP3 to phosphatidylinositol-4,5-bisphosphate (Oudit & Penninger 2009). PTEN was reduced during hypertrophic cardiomyopathy and correlated with several adaptive responses in cardiomyocytes (Crackower et al. 2002). However, mechanistic details of the modulatory activity of PTEN on Chagas disease have not been elucidated. To address this question, a cardiac cell line was infected with *T. cruzi* Berenice-62 (Be-62) strain and the expression of a specific set of microRNAs (miRNAs) was investigated. MiRNAs are an important class of endogenous, single stranded, small noncoding RNAs (~23-25 nt), which modulate gene expression and aid in the fine tuning of molecular mechanisms in a cell. Although, according to the miRBase (mirbase.org/Release21), more than 2,500 human miRNAs have been identified, the biological functions of only a small fraction have been characterised and, in *T. cruzi*-infected cells, such analyses have just begun. In our analyses, the miR-190b demonstrated a close modulatory connection with the control of PTEN expression, which collaborates with *T. cruzi*-infected cells viability.

## MATERIALS AND METHODS

*Cell culture* - H9c2 (2-1) cells [American Type Culture Collection (ATCC): CRL-1446] are an embryonic rat ventricular cell line, which were grown and maintained in Dulbecco’s modified Eagle’s medium (DMEM) supplemented with 10% fetal bovine serum (FBS) and 100 μg/mL penicillin/streptomycin under an atmosphere of 5% CO_2_ at 37ºC. All reagents were purchased from Life Technologies™, Brazil.

*Parasite infection* - *T. cruzi* (Be-62 strain, discrete typing unit II) was propagated in Vero cell monolayers (ATCC: CCL-81) in DMEM with 2% FBS and infective trypomastigotes were harvested as described previously (Petersen & Burleigh 2003). Next, 1.5 x 10^7^ parasites were incubated with 1.5 x 10^6^ H9c2 cells for 2 h at 37ºC under 5% CO_2_ to allow parasite-cell interaction. The remaining extracellular parasites were aspirated and the cells were extensively washed with phosphate-buffered saline (PBS) (2.7 mM KCl, 1.5 mM KH_2_PO_4_, 137 mM NaCl, and 8 mM Na_2_HPO_4_, pH 7.4); after which, fresh medium was added to the culture. Flasks containing infected cell cultures were immediately collected (0 h) or incubated for 2 h, 6 h, 12 h, 24 h, or 48 h, at which time-points, cells were collected. Uninfected cells were used as the control condition in our assays.

*Cellular viability assays and nucleus immunostaining* - Cellular viability was verified in assays using 1.5 x 10^4^ cells that had previously been grown in a 96-well plate. After 2 h of *T. cruzi*interaction, as previously described, cells were grown for up to 48 h and cellular viability was determined using methylthiazolyl diphenyl tetrazolium bromide in (2-methoxy-4-nitro-5-sulphophenyl)-2H-tetrazolium-5-carboxanilide (MTT) assay at specific time-points (Mosmann 1983). The plates were read at 570 nm using a microplate reader (Packard Instrument Company Inc, USA). In addition, to verify the effect of PTEN protein inhibition on cell viability, SF1670 (Sigma-Aldrich, USA) was added to cell cultures at a final concentration of 500 nM (Li et al. 2011). The cultures were incubated for up to 48 h and fresh media containing PTEN inhibitor was replaced at 24 h. Cellular viability was also determined at specific time-points and to verify the physiological effect of such inhibitor in the H9c2 cells at the very early time-point, SF1670 was added to them and subsequently incubated at 37ºC for 30 min (Li et al. 2011). For nucleus staining and microscopy analyses, 1.5 x 10^4^ cells were grown on coverslips and fixed in 3.8% paraformaldehyde containing 0.2% Triton X-100 (Sigma-Aldrich) for 7 min at 37ºC. Next, the nuclei were counterstained in a solution of 3.33 ng/mL of DAPI (Sigma- Aldrich) and subjected to microscopic analysis. Images were obtained with a Leica DMLB photomicroscope equipped with an HBO 100 W mercury lamp and the corresponding filter sets. The assays were performed in quadruplicate and statistical analyses were performed by counting 100 cells.

*RNA isolation and quantitative reverse-transcribed polymerase chain reaction (qRT-PCR)* - Total RNA was extracted using TRIzol^®^ Reagent (Life Technologies™) from 1.5 x 10^6^ cells either infected with *T. cruzi* or left uninfected. One microgram from each RNA sample was RT into first-strand cDNA using Cloned AMV Reverse Transcriptase (Life Technologies™), following the supplier’s instructions. All RT reactions were analysed by qRT-PCR using SYBR Green Master Mix (Life Technologies™) in an ABI 7300 Sequence Detection System (Applied Biosystems, USA). The relative expression levels of PTEN were normalised to *β-actin* gene and analysed by the 2-^ΔΔCT^method. For miRNA analyses, cDNA was prepared using Mini Script Reverse Transcription (Qiagen, Germany), which contains a special stem-loop primer for miRNAs. qPCRs were also performed using specific set of primers for miR-16-5p, miR-let7f-2-3p, miR-26b, miR-3586-3p, miR-190b, and the miScript SYBR Green PCR Kit (Qiagen) after extensive analyses of the 3’-UTR sequence of the*Pten* RNA sequence (Gene ID: 50557) from *Rattus norvegicus*. For that, miRBase search tool (mirbase.org) was used and putative miRNAs binding sequences were found in the analysed sequence. The 2-^ΔΔCT^ relative quantification method was applied and U6 was used as the internal control.

*Western blot (WB) analysis* - Whole cell extracts were prepared according to Sambrook et al. (2000). Equal amounts of protein (50 µg) were separated by electrophoresis in 10% polyacrylamide gels and then electrotransferred to polyvinylidene fluoride membranes. The membranes were immunoblotted overnight with rabbit anti-AKT and anti-phospho AKT, anti-PTEN, and anti-phospho-PTEN (P-PTEN), or rabbit anti-β actin (Cell Signaling, USA) polyclonal antibodies, followed by 2 h of incubation with a horseradish peroxidase-conjugated goat anti-rabbit antibody (Cayman Chemical, USA). Immunoreactive bands were visualised using a chemiluminescent detection kit (ECL^TM^; GE Healthcare, UK) and exposed to hyperfilm (GE Healthcare). The bands were quantified with Quantit One Software (Bio-Rad).

*Luciferase reporter assays* - The sequence corresponding to the 3’-UTR of Pten mRNA from 514-528 bp was cloned into the pGL3-Control vector (Promega) upstream of the firefly luciferase coding sequence *via*synthetic oligonucleotides ligation. This construction corresponds to the pGL3-PTEN plasmid. Next, 3 x 10^4^ cells were transiently transfected with 100 ng of pGL3-Control, or pGL3-PTEN along with 30 nM designed rno-miR-190b inhibitor, or a miRNA inhibitor negative control (Life Technologies™), and 20 ng of the internal control plasmid (pRL-TK vector, Promega, expressing Renilla Luciferase). For transfections, Lipofectamine 2000 reagent (Life Technologies™) was used. The activities of both luciferases were determined using Promega Dual-Luciferase Reporter Assay System (Promega) 24 h post-transfection, according to the manufacturer’s instructions. The relative luciferase activity was determined by normalisation with Renilla Luciferase activity and they were measured using TD20/20 luminometer (Turner Designs).

*miRNA inhibitor transfection and functional activity of miRNAs* - The functional activity of miRNAs, 3 x 10^4^ cells were transiently transfected with 30 nM of the designed rno-miR-190b inhibitor or the miRNA inhibitor negative control as previous described. After 24 h, cells were harvested for protein analyses and qPCR. In addition, the infection rates of rno-miR-190b inhibitor transfected-cells were investigated. For that, after 24 h of cellular transient transfection, 1.5 x 10^6^ cells were incubated with 1.5 x 10^7^ *T. cruzi* parasites to allow parasite-cell interaction for 2 h, as previously described. The viability was measured after 48 h post-infection (p.i.) in MTT assays. Uninfected and rno-miR190b inhibitor transient transfected-cells were used as control.

*Graphs and statistical analyses* - Values from three independent assays were used for the analyses and graphs were generated using Graph Pad Prism^®^ 5. The differences between the control and infected groups were also measured using one-way analysis of variance followed by Dunnett’s test. Significance was set at *p < 0.05, **p < 0.01, and ***p < 0.001.

## RESULTS

**Fig. 1:**
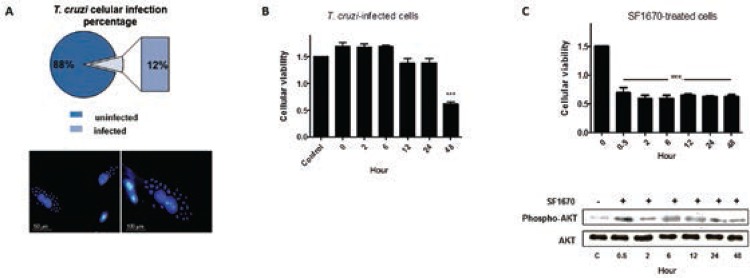
: biological effects of the *Trypanosoma cruzi* strain Berenice-62 infection in H9c2 cellular survival. Cells were grown and infected with the parasite and the initial 48 h after parasitic infection were investigated. A: infected cells were analysed*via* fluorescence microscopy; the nuclear material was labelled with DAPI (Bar = 50 μm and 100 μm). The representative rates of infected and uninfected cells are demonstrated in the circle; B: cellular viability assays after *T. cruzi* cellular infection; C: cellular and molecular analyses of the effect of the PTEN inhibitor (SF1670) in H9c2 cells. Cellular viability was measured by the formazan production and plotted in graphs. The western blot analyses demonstrated the effect of the drug in AKT phosphorylation. The presented values are the average from three independent experiments and the error bars represent the standard deviation of the mean. One-way analysis of variance testing showed significant differences between the control and cell samples and the significance level was set at p < 0.05 (***).

*T. cruzi* invasion and survival in host cells are critical steps in the establishment of infection. Based on these observations, we first investigated the efficiency of the parasite infectivity in our in vitro model. After 2 h of parasite-host cell interaction, approximately 12% of cells were infected. The infection was also analysed 48 h p.i. *via* fluorescence microscopy (Fig. 1A). Next, cellular viability of infected cells was examined at precise time-points after parasitic interaction (0 h, 2 h, 6 h, 12 h, 24 h, and 48 h), and uninfected cells were used as control in the assays. A 58.4% reduction in cellular viability at 48 h p.i. was observed (Fig. 1B). In addition, uninfected cells were treated with PTEN specific inhibitor (SF1670) and a reduction in cellular viability by ~50% (Fig. 1C) was observed, even after 30 min of PTEN activity inhibition, suggesting that the protein negatively modulates H9c2 cellular viability. The additional WB analyses reinforced the inhibitory effect of the drug on PTEN activity, which indirectly stimulates the phosphorylation of AKT protein (Fig. 1C). The inhibition of PTEN activity favours the accumulation of PIP3 in cell membrane, which binds to AKT in its pleckstrin homology domain (Haugh et al. 2000, Schmid et al. 2004, Li et al. 2011). Only the AKT molecules on the plasma membrane can be phosphorylated by protein kinases and become activated (Li et al. 2011). The analyses demonstrate that in the presence of SF1670, the phosphorylated AKT level considerably increased when compared to the untreated cells.

**Fig. 2:**
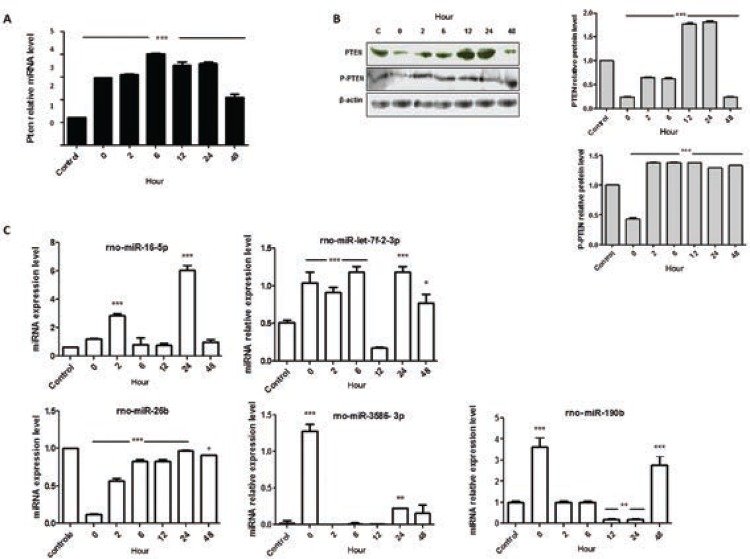
: modulatory effect of *Trypanosoma cruzi* in cellular metabolism of H9c2-infected cells. A: quantitative polymerase chain reaction analyses of Pten mRNA; B: Western blots analyses and quantification of PTEN, phospho-PTEN (P-PTEN), and the reference protein β-actin; C: miRNAs expression patterns in *T. cruzi-*infected cells. The presented values are the average from three independent experiments and the error bars represent the standard deviation of the mean. One-way analysis of variance testing showed significant differences between the control and cell samples and the significance level was set at p < 0.05 (***).

To investigate how PTEN could be possibly connected with the changes in cellular viability, mRNA expression and WB analyses were performed. In this acute cell culture model of infection, Pten mRNA significantly increased its expression level after cellular infection, reaching approximately 300% higher levels after 6 h of parasite infection (Fig. 2A). However, this mRNA expression pattern in *T. cruzi*-infected cells did not match with the PTEN protein expression. The WB analyses demonstrated that the PTEN protein levels decreased immediately after parasitic entry (0 h time-point) and increased considerably at 12 h and 24 h p.i., 77% and 81.09%, respectively, higher levels than the control. Moreover, despite the reduced functional activity in cellular system, the phosphorylated form of PTEN was investigated (Fig. 2B). A reduced level of the phosphorylated protein was also observed at 0 h p.i. (~57% lower than the control level) (Fig. 2B) and then its level increased its level to an average rate of 35% in the succeeding time-points. However, the absolute quantification of PTEN forms (data not shown) demonstrated a reduced phosphorylated form of the protein when compared to the nonphosphorylated form.

Next, to describe the fine-tuning that controls gene expression, five miRNAs that possibly modulate PTEN expression were investigated (Fig. 2C). The results suggested a potential connection between the miRNA-190b and PTEN protein expression pattern, considering a negative inverse correlation between the above two molecules. While the mRNA levels of Pten were found at higher levels in infected cells, the rno-miR-190b oscillated. Immediately after parasite entry into host cells, the miR-190b increased ~360%, returning to near basal expression level in the next two investigated time-points (2 h and 6 h). Next, the expression pattern of miR-190b at 12 h and 24 h time-point was almost abolished, which is an inversed correlation to the PTEN protein expression pattern at such time-points. At 48 h time-point, the miR-190b presented a higher expression level that was also an inversed correlation to the expression pattern of PTEN protein.

**Fig. 3:**
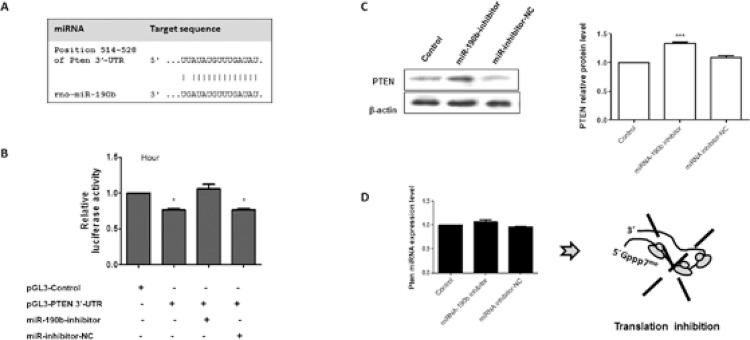
: molecular interplay between PTEN and miRNA-190b. A: schematic representation of the 3’-UTR sequence of Pten mRNA and the potential target region of the rno-miRNA-190b; B: relative luciferase activity in transfected cells with pGL3-Control and pGL3-PTEN-3’-UTR construct along with miRNA inhibitors. The results were plotted on representative graphs. The empty expression vector (pGL3-Control) was used to maintain an equivalent amount of DNA inside the cells and to express a basal level of luciferase; C: Western blots analyses and quantification of PTEN and the reference protein β-actin after transfection of miRNA inhibitors followed by quantitative polymerase chain reaction analyses. A translational blockage model is schematically represented. Data are representative from at least three independent experiments, p < 0.05 (***); NC: negative control.

To confirm the mechanistic correlation between the miRNA-190b and PTEN, the potential target site at the 3’-UTR of Pten mRNA (514-528 bp) and the miRNA-190b were cloned and used in cellular transfection. The potential target site at the 3’-UTR of Pten mRNA and the miRNA-190b expression pattern is demonstrated in Fig. 3A. Next, luciferase assay followed by WB and qPCR analyses were performed. For luciferase assays (Fig. 3B), transfected pGL3-Control cells produced basal luciferase activity and the plasmid was used to maintain an equivalent amount of DNA inside the cells. On the other hand, the presence of pGL3-PTEN in H9c2 cells demonstrated the modulatory effect of the 3’-UTR sequence of PTEN in the luciferase production. A reduction of ~22% luciferase production was observed when compared with the expression level of the protein by the control vector (pGL3-Control). In addition, the presence of the miR-190b inhibitor or its negative control reinforces the existence of a physical interaction between this miRNA and the Pten 3’-UTR. When cells were co-transfected with the pGL3-PTEN along with the specific inhibitor, the expression level of luciferase returned to near the basal level after 24 h transfection. The physical binding between the miR-inhibitor and miR-190b molecules decreased the small interfering RNA activity on PTEN-3’-UTR mRNA and facilitated the production of luciferase (Fig. 3). In addition, the co-transfection assays performed with the miR-inhibitor negative control did not modulate the pattern of luciferase production in pGL3-PTEN-transfected cells.

Next, the effects of miRNA-190b inhibitor and its negative control were tested in WB and qPCR assays. The results demonstrated that the presence of specific miRNA-190b inhibitor increased the protein level by 40%, and the miRNA inhibitor negative control did not change the PTEN protein level when compared to the nontransfected culture in the 24 h investigation (Fig. 3C). The qPCR analyses reinforced the observation that the miRNA-190b blocks the Pten mRNA translation (Fig. 3C), considering the presence of very small changes in Pten mRNA pattern of expression under the investigated conditions.

**Fig. 4:**
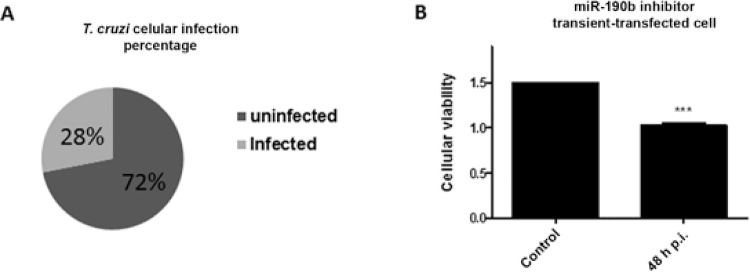
: biological effects of the *Trypanosoma cruzi* strain Berenice-62 infection in H9c2 miRNA-190b inhibitor transient transfected. Cells were grown, transient transfected and infected with the parasite. A: infected cells were counted and the percentage rates of infected and uninfected cells are demonstrated in the circle; B: cellular viability assays of miRNA-190b inhibitor transient transfected-cells infected or uninfected with the *T. cruzi* after 48 h post-infection (p.i.) were represented in the graph. The presented values are the average from three independent experiments and the error bars represent the standard deviation of the mean. One-way analysis of variance testing showed significant differences between the control and cell samples and the significance level was set at p < 0.05 (***).

Finally, to evaluate the physiological effect of the miRNA-190b inhibitor in*T. cruzi* infection of H9c2 cells, transient transfected-cells were infected with the parasite and infection rate and cellular viability were also measured. Fig. 4 presented the results and the presence of miR inhibitor facilitated entry of the parasite, which raised the percentage of infected cells (Fig. 4A). An average infection rate of ~28% was observed in the three independent assays performed. Moreover, cellular viability measured in MTT assays also increased in the miRNA-190b inhibitor transient transfected-cells, when they were infected with the parasite. At 48 h time-point (Fig. 4B) p.i., the viability rate reduced ~31.8% when compared to the uninfected and transient transfected-cells with the miR inhibitor. The combined results reinforced the negative functional activity of the miRNA-190b in *T. cruzi-*infected cells survival.

## DISCUSSION

Several studies have demonstrated that *T. cruzi* induces multiple responses in the heart that are necessary for the successful establishment of infection (Burleigh 2005, Yoshida 2006, Mott et al. 2009); however, the mechanistic understanding of this pathological process remains the focus of scientific investigations. In this study, we investigated PTEN in *T. cruzi* Be-62-infected H9c2 cells, considering its direct connection in controlling the AKT/pKB molecular pathway, which modulates cellular growth, hypertrophy, and even death of infected cells.

To understand the modulatory effect of the parasite in cellular survival during the onset of infection, cellular viability was measured and considerable reduction in it was observed at 48 h time-point (Fig. 1B). PTEN was addressed as one important element correlated with the control of cellular viability. Despite the low parasite infection rates of H9c2 cells (Fig. 1A), it has been well described that*T. cruzi* infection induces global host cell response in cardiomyocytes (Petersen & Burleigh 2003,Manque et al. 2011), due to the cellular mediators produced by infected cells. In our cellular model, PTEN protein level was measured, and the increased amount of PTEN at 12 h and 24 h post-interaction (Fig. 2B) seems to correlate with a down-regulated expression of miR-190b (Fig. 2C), instead of the Pten mRNA expression level (Fig. 2A), which is maintained at higher level all along the investigated time-points. In addition, the high relative levels of P-PTEN in the assay, when compared to the phosphorylated protein in uninfected culture, reinforce the observation that this molecular pathway is strictly regulated to assure homeostasis. It was well described that, despite the reduced functional activity in the cellular system, the phosphorylated form of PTEN is more stable (Vazquez et al. 2000), which could be more convenient in a stressed biological system, where many players act to avoid apoptosis.

In fact, the complexity of PTEN activity and function in cellular systems has many details. In this scenario, miRNAs are small molecules that are able to modulate gene expression at the post-transcriptional level by targeting mRNA for degradation or by repressing the translation of mRNAs (Nilsen 2007). Based on these observations, we checked the expression level of five miRNAs and the miR-190b seems to be involved in PTEN regulation (Fig. 2C).

Despite the asynchronous expression of this miRNA and Pten mRNA, the combined results suggest that this molecule could be a relevant player in the down regulation of PTEN protein at 0 h of parasitic infection. To validate this observation, the putative binding site of the miR-190b at the 3’-UTR sequence of PTEN from *R. norvegicus* was cloned into the pGL3 vector and transfected into H9c2 cells along with the corresponding miRNA inhibitor (miR-190b). The physical interaction between the molecules (mRNA and miRNA) was verified. The pGL3 vector contains the SV40 promoter and enhancer sequences, which results in the strong expression of luciferase in mammalian cells, and it is useful for monitoring RNA degradation, or translation blockage based on the reduction in luciferase activity; the finding was corroborated by WB analysis using miRNAs inhibitors. The analyses of PTEN protein after 48 h transfection with miR-inhibitors (specific and negative control) modulated the PTEN protein level, but did not affect significantly the Pten mRNA level.

In conclusion, it is known that parasite infection modulates cellular behaviour to ensure *T. cruzi* survival. However, in the investigated early stage of *T. cruzi* Be-62-infected cells, the reduced expression level of PTEN, by the modulatory post-transcriptional process of miRNA-190b on the messenger RNA, results in decreased cellular viability rates, which negatively contributes to the establishment of the infection. To reinforce such observations, our in vitro functional assay demonstrated increased rates of cellular infection and viability in H9c2 transient transfected cells with the rno-miR-190b inhibitor, when compared to the nontransfected cells. This finding corroborates the physiological negative function of the miR-190b in contributing to the survival of *T. cruzi*-infected cells. Probably, in vivo the parasite is able to evade this initial decrease in cellular viability and survives. Further assays will answer this question.
